# Gait Stride Length Estimation Using Embedded Machine Learning

**DOI:** 10.3390/s23167166

**Published:** 2023-08-14

**Authors:** Joeri R. Verbiest, Bruno Bonnechère, Wim Saeys, Patricia Van de Walle, Steven Truijen, Pieter Meyns

**Affiliations:** 1 Department of Sciences and Technology, Karel de Grote (KdG) University of Applied Sciences and Arts, 2660 Antwerp, Belgium; 2 REVAL Rehabilitation Research Center, Faculty of Rehabilitation Sciences, Hasselt University, 3590 Diepenbeek, Belgium; 3 Technology-Supported and Data-Driven Rehabilitation, Data Science Institute, Hasselt University, 3590 Diepenbeek, Belgium; 4 Department of Rehabilitation Sciences and Physiotherapy, MOVANT, Faculty of Medicine and Health Sciences, University of Antwerp, Wilrijk, 2610 Antwerp, Belgium; 5 Clinical Gait Analysis Laboratory Antwerp, Heder, Ekeren, 2180 Antwerp, Belgium

**Keywords:** embedded machine learning, tinyML, machine learning, regression, neural network, healthcare, gait analysis, gait stride length, inertial measurement unit, IMU, microcontroller, MCU, wearable sensors

## Abstract

Introduction. Spatiotemporal gait parameters, e.g., gait stride length, are measurements that are classically derived from instrumented gait analysis. Today, different solutions are available for gait assessment outside the laboratory, specifically for spatiotemporal gait parameters. Such solutions are wearable devices that comprise an inertial measurement unit (IMU) sensor and a microcontroller (MCU). However, these existing wearable devices are resource-constrained. They contain a processing unit with limited processing and memory capabilities which limit the use of machine learning to estimate spatiotemporal gait parameters directly on the device. The solution for this limitation is embedded machine learning or tiny machine learning (tinyML). This study aims to create a machine-learning model for gait stride length estimation deployable on a microcontroller. Materials and Method. Starting from a dataset consisting of 4467 gait strides from 15 healthy people, measured by IMU sensor, and using state-of-the-art machine learning frameworks and machine learning operations (MLOps) tools, a multilayer 1D convolutional float32 and int8 model for gait stride length estimation was developed. Results. The developed float32 model demonstrated a mean accuracy and precision of 0.23 ± 4.3 cm, and the int8 model demonstrated a mean accuracy and precision of 0.07 ± 4.3 cm. The memory usage for the float32 model was 284.5 kB flash and 31.9 kB RAM. The int8 model memory usage was 91.6 kB flash and 13.6 kB RAM. Both models were able to be deployed on a Cortex-M4F 64 MHz microcontroller with 1 MB flash memory and 256 kB RAM. Conclusions. This study shows that estimating gait stride length directly on a microcontroller is feasible and demonstrates the potential of embedded machine learning, or tinyML, in designing wearable sensor devices for gait analysis.

## 1. Introduction

Spatiotemporal gait parameters refer to the measurements obtained from the analysis of the walking pattern, which includes the gait stride length parameter. Gait analysis is crucial in clinical applications such as physical medicine and rehabilitation. Usually, it provides information about movements (kinematics); the forces involved in moving (kinetics), gait parameters related to the size and timing of the gait pattern (spatiotemporal parameters); and sometimes the muscle activity (electromyography). It provides a comprehensive understanding of an individual’s walking ability, including their gait pattern, speed, and balance. In addition, it allows for the identification of abnormalities or deviations from typical gait patterns, which can aid in diagnosing and treating various conditions, including neurological disorders, musculoskeletal injuries, and developmental disorders. Gait analysis can also help to monitor the effectiveness of treatment interventions, providing valuable information for optimizing therapy and rehabilitation programs [1].

Today, there are different systems available for instrumented gait analysis. However, these systems are expensive laboratory setups which mainly rely on multiple cameras. Therefore, these systems cannot be used outside of the laboratory. For spatiotemporal gait parameters, more specifically, solutions do exist for gait assessment outside of the laboratory. Such a solution is often a wearable device that comprises a sensor, an inertial measurement unit (IMU), and a processing unit (microcontroller, MCU). For example, the wearable Gait Up Physilog^®^ sensor [2] combined with the GaitUP LAB software can perform gait assessment. The Xsens DOT [3] is a wearable IMU sensor platform used to develop mobile motion analysis applications.

However, these existing wearable sensor devices are resource-constrained. They contain processing units with limited processing and memory capabilities. Furthermore, they are battery-powered and have low battery capacity; therefore, continuous sending of raw sensor data for an extended period will result in fast battery depletion. The solution which we propose is to perform data analytics and data processing locally on the wearable device. However, designing these data processing and efficient energy algorithms is a significant challenge for gait analysis [4]. Our proposed solution includes embedded or tiny machine learning (tinyML) [5,6].

Spatiotemporal parameters can be estimated by a machine or deep learning model [7,8,9,10,11]. Although the memory footprint of a deep learning model during inference is smaller than that while training the model, it can still lie outside of a processor’s available memory. Therefore, today’s challenge is the development of accurate machine and deep learning models for deployment on resource-constrained embedded devices. The design of these models requires a different design approach; namely, creating embedded machine learning or deep learning models is a trade-off between memory, latency, energy requirements, and model accuracy. These requirements must be considered at the initial phase of the design process. This article aims to design a machine learning model for gait stride length estimation which is deployable on a microcontroller.

A multilayer 1D convolutional neural network model is presented herein for gait stride length estimation using IMU sensor data in combination with a microcontroller as the processor. The proposed model can be deployed on a microcontroller, i.e., a Cortex-M4F 64 MHz with 1 MB Flash and 256 kB RAM. The model, the data and machine learning pipeline, and the state-of-the-art software tools (libraries and frameworks) which were utilized are explained in detail. In this article, a proof of concept is provided which indicates that accurate models for deployment on resource-constrained embedded devices are feasible. The results are directly relevant to the development of new wearables in gait analysis.

## 2. Related Work

This study uses the convolution neural network (CNN) topology as the model architecture with which to design a machine learning model to achieve gait stride length estimation for the purpose of gait analysis. A deployable model on a microcontroller, using relevant clinical gait data from an IMU sensor, was utilized.

Hannink et al. [9] described two CNN models which were used to extract eight spatiotemporal gait parameters. In the study, they obtained measurements from 99 geriatric inpatients using a free walking test at a comfortable speed. The IMU sensors were attached laterally below the ankle joint on both the left and right feet, and the data were captured at a sample rate of 102.4 Hz. The data split was performed depending on the patient identifier. The data pre-processing consisted of extracting the annotated stride (from heel strike to heel strike), sensor calibration, and coordinate system transformation. Before feeding the data into the neural network, the signals were normalized to the respective sensor range and zero padding to a fixed length of 256 samples were performed. Two deep-learning models were built. Model A estimated the complete set of output variables with a combined model, and Model B comprised an ensemble of smaller networks. The model architecture for model A consisted of three convolutional layers with max-pooling, followed by three densely connected layers and a readout layer. Model B consisted of two convolutional layers with max-pooling, one dense layer, and a readout layer. The study demonstrated that, according to the unseen test data, that the mean accuracy and precision for the stride length was −0.34 ± 8.10 cm for model A and −0.15 ± 6.09 cm for model B. The model performance evaluation for the two models was based on a 10-fold cross-validation scheme, and as a reference, the GAITRite was used. During the design of both models, no hyperparameter tuning was performed.

Hannink et al. [10] described a deep convolution neural network (DCNN) for stride length estimation in their geriatric analysis. The dataset was the same as that described in [9]; however, data from 101 patients were taken for the neural network design. The data split was performed depending on the patient identifier, and the same pre-processing steps were performed as those described in [9]. The trained network consisted of two convolutional layers and one dense layer. The study showed a mean accuracy and precision of 0.27 ± 5.43 cm for the stride length; the evaluation was based on a 10-fold cross-validation scheme; and the GAITRite was used as a reference. In the study, no hyperparameter tuning was performed.

Zrenner et al. [11] described a DCNN. This model was trained on 27 (amateur) runners. The subjects were asked to run at different velocities. As IMU, an in-house developed sensor was used, and the IMU data were captured at a sample rate of 200 Hz. The sensors were located in the midsoles of the running shoe. Gait segmentation was performed before the data were fed to the deep learning network; no extra signal processing was performed. The deep learning model was a modified version described in [8]. It consisted of two convolutional layers, two max pool layers, one flatting layer, one dense layer, and a readout layer. The authors also noted that they increased the number of epochs and modified the batch size. However, this did not improve the model performance nor its generalization. Apart from increasing the number of epochs and changing the batch size, no further hyperparameter tuning was performed. The mean accuracy and precision of the stride length was 1.3 ± 19.4 cm, and the evaluation was based on leave-one-subject-out cross-validation. This study used a motion capture system (Vicon Motion Systems Inc, Oxford, UK) as a gold standard for gait stride length.

Hannink et al. and Zrenner et al. showed that a CNN model combined with IMU data was able to estimate the gait stride length. However, embedded machine learning was not applied in any of the three studies.

## 3. Materials and Methods

### 3.1. Solution for an Intelligent Gait Monitoring System

**Figure 1:**
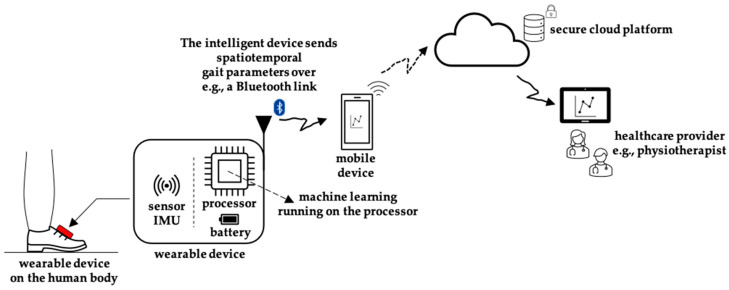
Design of our solution of an intelligent wearable device inside a gait-monitoring healthcare system.

Figure 1 illustrates our proposed solution for an intelligent gait monitoring system for gait analysis outside the lab environment using embedded machine learning. A wearable device attached to the human body would estimate the spatiotemporal parameters locally on the device, and then send these parameters to, e.g., a mobile device. Optional spatiotemporal parameters would be sent to a secure Cloud platform and shared with the healthcare provider. This article focuses on the design of the embedded machine-learning model for gait stride length estimation, deployable on a microcontroller processor located inside a wearable device.

### 3.2. Dataset

The study presented in this article used the data from the TRIPOD—**Tr**eadmill, **I**MU, **P**edobarographic and Ph**o**toelectric **D**ataset [12] study, available upon request for scientific research purposes. The measurements were obtained from 15 young and healthy participants (8 males, 7 females) who walked on a treadmill for two minutes at three different speeds (Table 1). After two minutes, the treadmill slowed down until it stopped. The dataset also included data from seven IMU sensors (Physilog^®^ 5 IMUSs from Gait Up, Lausanne, Switzerland). The sensors were located on the left and right instep, left and right heel, left and right shank, and one at the sacrum. The data were acquired at a sample rate of 128 Hz. The IMU data for the three different walking speeds are provided in a CSV file (RF.csv).

Additionally, one file (SyncInfo.csv) contains the timestamp of the initial contact. The ground truth was measured by a pressure-sensitive Zebris (zebris Medical GmbH, Isny, Germany) and photoelectric OptoGait (Microgate, Bolzano, Italy) system. In the multilayer 1D convolutional neural network design, only data from the IMU sensor attached to the right foot, instep position, were used. As ground truth, we used the stride length from the OptoGait system, which is provided in a CSV file (optogait.csv).

### 3.3. Data Pre-Processing and Machine Learning Workflow

**Figure 2:**
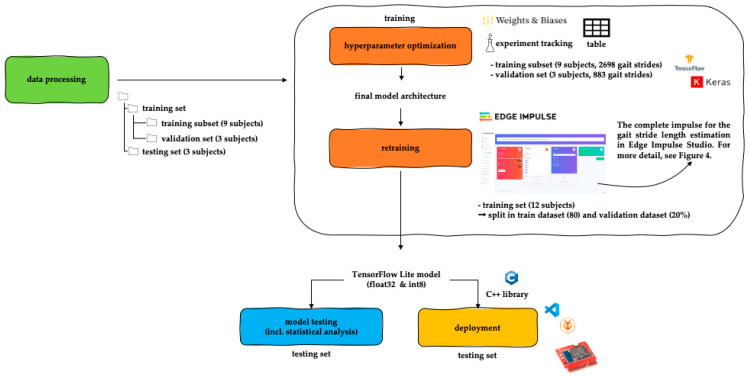
The data processing and machine learning workflow.

The model development workflow (Figure 2) consists mainly of 4 parts: data processing, model training, model testing (including statistical analysis), and deployment.

#### 3.3.1. Data Processing

While constructing the labeled dataset, we only used the first two minutes of the IMU data, starting from the first estimated initial contact. Different pre-processing steps were performed before the labeled sensor data was applied to the multilayer convolutional neural network. The data pre-processing was written in Python 3.10 [13] and SciPy v.1.9.3 [14]. The following pre-processing steps were performed:

- Normalize. The signals from the gyroscope and accelerometer were normalized to the respective sensor ranges. The sensor range was 16 g for the accelerometer [12] and 1000°/s [12] for the gyroscope.
- Resampling. In this study, we developed a model for an IMU signal sampled at 60 Hz; the data were down-sampled to 60 Hz.
- Estimation Initial Contact (IC). The triaxial accelerometer and the triaxial gyroscope data were fed to a second-order low-pass Butterworth filter with a cut-off frequency of 10 Hz [15]. Zero-phase filtering was applied to preserve the features of the waveform. Using the detection algorithm described by Maqbool et al. [15], the initial contact (IC) time was estimated.
- Gait cycle segmentation and labelling. Gait cycle segmentation was performed based on the estimated IC time. Next, the data were labeled and stored in a JSON format [16]. Additionally, the first measured gait cycle was removed for each trial to avoid potential artefacts due to motion initiation.

The pre-processing of the data resulted in a dataset of 4467 gait strides. The dataset was divided, depending on the participants’ identifier (SUB <xx>), into a training set (12 subjects, 3581 gait strides) and a testing set (3 subjects, 886 gait strides).

#### 3.3.2. Training

The model training consisted of two phases. First, hyperparameter optimization was performed. We used the machine learning operation (MLOps) platform Weights & Biases, wandb version 0.12.20 [17], for experiment tracking. The training was performed on a 64-bit Intel^®^ Core™ i9-10900K CPU (20 cores), 3.70 GHz, 128 GB RAM, and an NVIDIA GeForce RTX3080 GPU type. The OS which we utilized was Ubuntu 20.04.5 LTS. The TensorFlow (version 2.10.0) deep learning framework, using the Keras high-level API [18], was used in this study.

**Figure 3:**
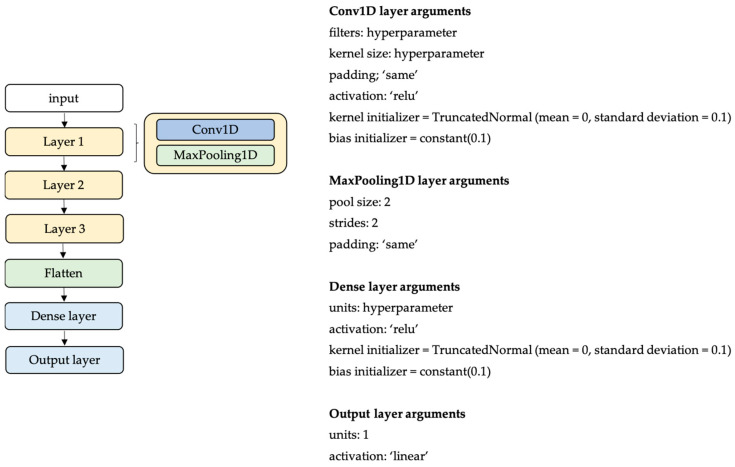
Model architecture.

The model architecture, Figure 3, consists of three 1D convolutional (Conv1D) and max-pooling layers, followed by a fully connected layer (Dense Layer) and an output layer. For optimization, the Adam optimizer was used (β_1_ = 0.9, β_2_ = 0.999 and ε = 1e^−8^). The number of epochs was set to 1500. Kernel initialization (truncated normal, mean = 0, stdev = 0.1) and bias (constant = 0.1) initialization were applied to the Conv1D layer and the dense layer, respectively. The Conv1D filters and kernel size, the number of units for the dense layer, and the learning rate and batch size were the hyperparameters in the model design (Table 2). As a search strategy, the grid search method was applied. The training and validation mean squared error as well as the model parameters, filters, kernel sizes, units, learning rates, and batch sizes were logged and summarized in a table on weights and biases.

Hold-out cross-validation was used during hyperparameter tuning. Therefore, the training set (12 subjects, 3581 gait strides) obtained during the data processing step was further divided depending on the participants’ identifiers (SUB <xx>), into a training subset (9 subjects, 2698 gait strides) and a validation set (3 subjects, 883 gait strides). Before the data were fed into the neural network, zero padding was applied, fixing the signal length for each sensor axis to 108 samples.

**Figure 4:**
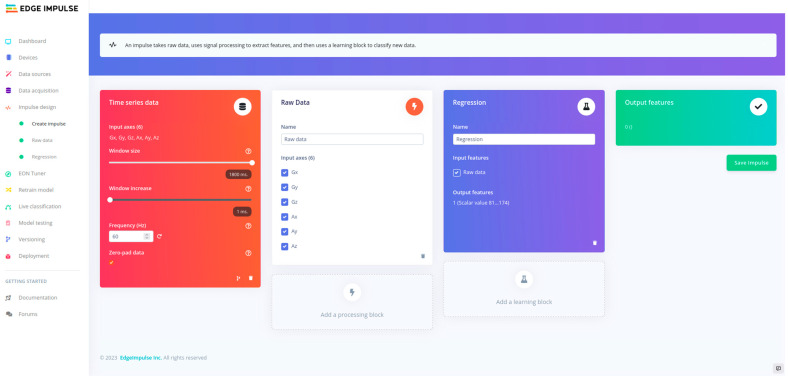
The complete impulse for the gait stride length estimation in Edge Impulse Studio.

After ending the hyperparameter tuning, the model was retrained using the cloud-based MLOps platform Edge Impulse Studio [19] (Figure 4) (Edge Impulse, San Jose, CA, USA). The projects in Edge Impulse studio were divided into a series of blocks representing the data flow. The different blocks combined resulted in an impulse. Figure 4 shows the workflow, in Edge Impulse Studio, with the various blocks. The left block, “Time series data”, represents the labeled data. The window size was set to 1.8 sec, and zero padding was applied. The zero padding fixed the length for each axis to the same signal length (108 samples) used during the hyperparameter tuning phase. The middle block was the processing block. The raw data processing block was selected in this study. The scale axis in the processing block was set to one because normalization was already applied during the data pre-processing step. The output of the raw data processing block provided data without pre-processing or signal processing being applied. The learning block followed the raw data processing; a regression learning block was selected. The training settings and neural network architecture were defined in the learning block.

During the retraining phase in Edge Impulse Studio, the training set (12 subjects, 3581 gait strides) obtained during the data processing step was divided into a ‘train dataset’ (80%) and a ‘validation dataset’ (20%). No further model optimizations, i.e., hyperparameter tuning, were performed. The retraining in Edge Impulse Studio resulted in a TensorFlow Lite model transformed into a float32 and a quantized int8 model. The int8 model required a representative dataset for its quantization [20]. In this study, the validation dataset was used as the representative dataset.

#### 3.3.3. Model Testing and Statistical Analysis

Given the testing set (from the three subjects, 886 gait strides) obtained during the data processing step, the stride length was predicted using the float32 and the int8 models. This testing set was never used during hyperparameter optimization nor the retraining phase in Edge Impulse Studio. The Band–Altman plot [21] assessed the float32, the int8 model prediction, and the ground truth agreement. The bias (mean error), the upper and lower limits of agreement (LoA), and the minimal detectable change (MDC) were calculated [22]. Additionally, the mean square error (MSE) and R-squared (R^2^) were provided.

#### 3.3.4. Deployment

Edge Impulse Studio provides optimized source code libraries. This source code library can be further customized and integrated into an application. In this study, we selected the Arduino library and used PlatformIO [23] (Core 6.1.6, Home 3.4.3) to build the firmware. The embedded platform which we selected and used for evaluation was the SparkFun MicroMod with an nRF52840 Processor Board (v1.0) [24] mounted on a SparkFun MicroMod Data Logging Carrier Board (v1.2) [25] (SparkFun, Colorado, USA). The nRF52840 was an Arm^®^ Cortex^®^-M4F 64 MHz processor from Nordic Semiconductor, with 1 MB internal flash and 256 kB internal RAM.

**Figure 5:**
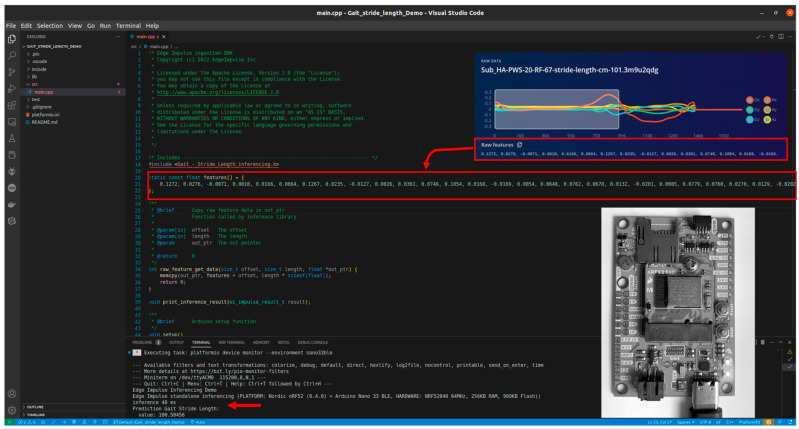
The static buffer code snippet, using Edge Impulse C++ SDK.

We used the static buffer implementation example from Edge Impulse (Figure 5) to test the model on the device. In this implementation, the raw features from one gait cycle were copied into a static buffer. These features were fed to the input neural network. This implementation allowed us to test the model inference without connecting a microcontroller to an actual IMU sensor. Using Edge Impulse Studio, an estimation of memory usage (RAM and flash) and latency was performed. Afterwards, the PlatformIO tool was used to estimate the RAM and Flash memory of the deployed model on the nRF52840 processor.

## 4. Results

### 4.1. Training—Hyperparameter Tuning

The hyperparameter tuning resulted in an optimal model for the given TRIPOD dataset. The outcome of the hyperparameter tuning showed a batch size of 64 and a learning rate of 0.0001; the obtained filter and kernel size for the three Conv1D layers and the units for the dense layer are shown in Table 3.

### 4.2. Retraining Using Edge Impulse Studio and Deployment

**Figure 6:**
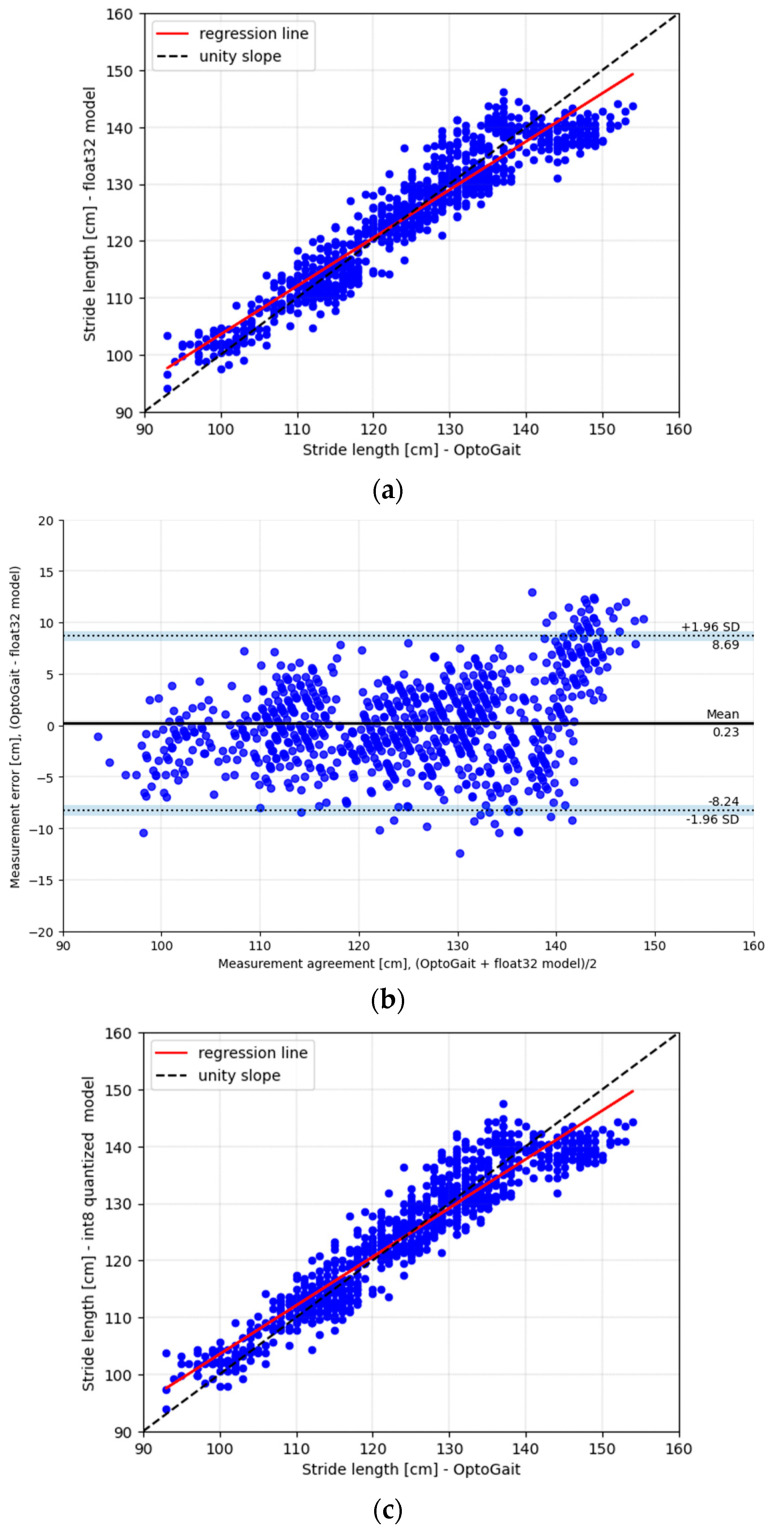

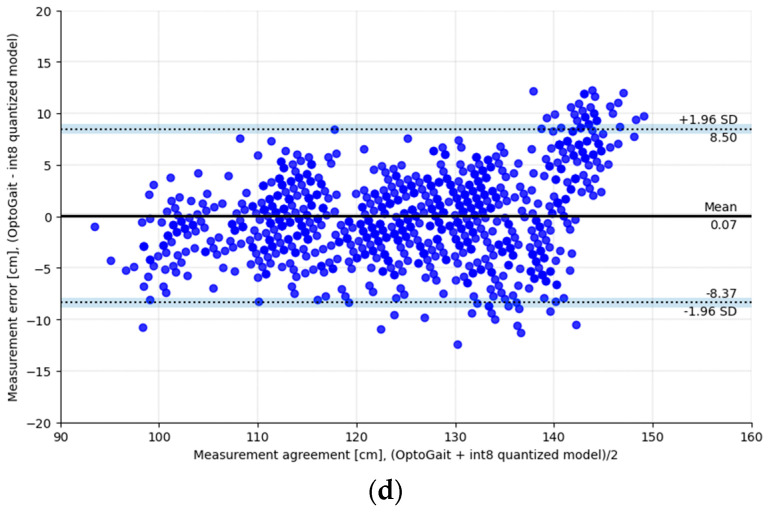
float32 vs. int8. (**a**) Comparison of the estimated and the measured stride length from the float32 model and the OptoGait; (**b**) Bland–Altman plot for the stride length, estimated by float32 on the testing set (**c**) comparison of the estimated and the measured stride length from quantized int8 model and the OptoGait; (**d**) Bland–Altman plot for the stride length estimated by int8 on the testing set.

The model architecture (Figure 3), using the model parameters shown in Table 3, was retrained and tested using Edge Impulse Studio. For model evaluation, we used a testing set of 3 subjects (886 gait strides). The testing set was never used during hyperparameter optimization nor the retraining phase in Edge Impulse Studio. Table 4 shows the calculated MSE, limits of agreement, mean error, and R^2^. Figure 6 compares the estimated and the measured stride length. Additionally, the Bland–Altman plot for the stride length estimated for float32 and the int8 is provided. Table 5 shows the memory estimate by Edge Impulse Studio for float32 and the int8 model, Table 6 shows the model performance using leave-one-out cross-validation, and Table 7 shows the memory estimation by the PlatformIO tool.

## 5. Discussion

This study aimed to create a model design for gait stride length estimation that could be deployed on a microcontroller. The final model architecture consisted of three 1D convolutional (Conv1D) and max-pooling layers, followed by a fully connected layer (dense layer) and an output layer (Figure 3). The outcome of the hyperparameter tuning resulted in a model architecture with model parameters as presented in Table 3.

In this study, leave-one-out cross-validation was used to evaluate the model’s performance, Table 8. Compared to Zrenner et al. [11], this led to an improvement in standard deviation: 8.35 cm vs. 19.4 cm (Table 8). It must be noted, however, that the dataset used in the study of Zrenner et al. comprised data from runners running overground, and no hyperparameter tuning was performed. Compared to Hannink et al. [9,10], the multilayer 1D convolutional model presented in this study still had a slight bias of 1.7 cm and a higher standard deviation. However, making a fair comparison was difficult due to differences in the dataset (geriatric inpatients vs. healthy subjects) and in the model performance evaluation (10-fold cross-validation vs. leave-one-out cross-validation). For future research, a more standardized model performance comparison for embedded machine learning will be needed in order to make a fairer comparison.

It should be emphasized that unlike the studies of Zrenner et al. and Hannink et al., the model presented herein needed to be deployed on a resource-constrained device. Therefore, during the model’s optimization and the selection of the final model, not only does MSE need to be taken into consideration, but the memory and latency requirements must also be examined.

The model’s architecture (Figure 3), with its parameters provided in Table 3, was retrained in Edge Impulse Studio, resulting in a float32 and int8 quantized model. Quantization to an int8 model results in a smaller model size, occupying less memory and decreasing inference time. Quantization can potentially reduce the accuracy of the model. However, in the present study, as shown in Table 4, for the given model architecture, model parameters, and dataset, no MSE decrease was observed between the float32 and the quantized int8 model. The float32 model had a mean error of 0.23 ± 4.3 cm, a memory usage of 284.5 kB flash and 31.9 kB RAM, and an inference time of 769 ms. The int8 model had a mean error of 0.07 ± 4.3 cm, a memory usage of 91.6 kB flash and 13.6 kB RAM, and an inference time of 80 ms. Various pre-processing steps were required before the data were fed to the multilayer convolutional neural network. In this study, the pre-processing block, consisting of filtering, initial contact detection, and gait segmentation, was not implemented on the embedded devices and, therefore, was not included in the latency or memory calculations.

Both models were able to be deployed on the nRF52840, an Arm^®^ Cortex^®^-M4F 64 MHz processor from Nordic Semiconductor, with 1 MB internal flash and 256 kB internal RAM. The memory consumption estimated by PlatformIO (Table 7) was higher than the values shown in Table 5. This difference was caused by the necessary code dependencies.

## 6. Study Limitations

The study’s main limitation was the limited available data. The TRIPOD dataset consisted of only 15 young (average age of 26.4 ± 3.7 years) healthy participants (8 males, 7 females) walking on a treadmill. The pre-processing of the data resulted in a dataset of 4467 gait strides which was divided into a training set (12 subjects, 3581 gait strides) and a testing set (3 subjects, 886 gait strides). In order to estimate the performance, only a limited portion of the dataset was used for testing; it consisted of a limited number of gait strides (886) from only three subjects. Moreover, we did not investigate the generalization of the model to a different clinical population.

## 7. Conclusions

In this study, we aimed to create an embedded machine-learning model for gait stride length estimation that could be deployed on a microcontroller. A float32 and int8 multilayer 1D convolutional neural network is presented. The float32 model developed herein demonstrated a mean accuracy and precision of 0.23 ± 4.3 cm, and the int8 model showed a mean accuracy and precision of 0.07 ± 4.3 cm. For the given model’s architecture, parameters, and dataset, no MSE decrease was observed between the float32 and the quantized int8 model. The memory usage for the float32 model was 284.5 kB flash and 31.9 kB RAM. The int8 model memory usage was 91.6 kB flash and 13.6 kB RAM. Both models were able to be deployed on a microcontroller. In this study, the nRF52840, an Arm^®^ Cortex^®^-M4F 64 MHz processor from Nordic Semiconductor with 1 MB internal flash and 256 kB internal RAM, was selected as the processor for evaluation.

The study’s outcome shows that a gait stride length estimation directly on a resource-constrained embedded device is feasible and demonstrates the potential of embedded machine learning in designing wearable devices for gait analysis. However, to unlock the full potential of embedded machine learning for gait analysis, we need more standardized clinical datasets from different populations which are measured by various IMU sensors and at different on-body locations. Additionally, a more standardized method of comparing the performances of embedded machine learning techniques is required in order to make a fairer comparison of models, given their training datasets.

## Figures and Tables

**Table 1:** TRIPOD dataset sample characteristics (Table 2 in reference [12]) (SD: standard deviation, PWS: preferred walking speed on the treadmill).

	Minimum	Mean ± SD	Maximum
Age (y)	20	26.4 ± 3.7	34
Mass (kg)	53.5	69.7 ± 12.1	103
Height (cm)	157.5	176.2 ± 8.8	190
Leg length ^1^ (cm)	76	86.2 ± 4.3	95
PWS (km/h)	3.1	3.9 ± 0.5	4.9

^1^ between the greater trochanter and lateral malleolus.

**Table 2:** Grid search parameter values.

	Parameter Value
batch size	{32, 64, 128, 256}
Adam—learning rate (α)	{0.001, 0.0001, 0.00001}
Conv1D filters	{16, 32}
Conv1D kernel size	{5, 15}
Dense units	{128, 256, 512, 1024}

**Table 3:** Model parameters (batch size = 64, learning rate = 0.0001, epochs = 1500).

	Parameters
Conv1D Layer 1 filter	32
Conv1D Layer 1 kernel size	5
Conv1D Layer 2 filter	16
Conv1D Layer 2 kernel size	5
Conv1D Layer 3 filter	32
Conv1D Layer 3 kernel size	15
Dense Layer units	128

**Table 4:** Statistical analyses: float32 vs. int8 model (testing dataset).

	float32	int8
ME ± stdev	0.23 ± 4.3 cm	0.07 ± 4.3 cm
Upper LoA	8.69 cm	8.5 cm
Lower LoA	−8.24 cm	−8.37 cm
MDC	8.46 cm	8.43 cm
MSE	18.7	18.5
R^2^	0.89	0.89

**Table 5:** Memory (RAM and flash) and latency, float32 vs. int8 model—provided by Edge Impulse studio using EON™ Compiler, an estimate for the nRF52840 (testing dataset).

	float32	int8
RAM	31.9 kB	13.6 kB
Flash	284.5 kB	91.6 kB
Latency	769 ms	80 ms

**Table 6:** Model’s performance using leave-one-out cross-validation.

ME ± stdev	Population and Methods
1.07 ± 8.35 cm	healthy subjects—treadmill walking ground truth for stride length: Optogait

**Table 7:** Memory (RAM and flash) and latency, float32 vs. int8 model—the memory is an estimate provided by PlatformIO.

	Available	float32	int8
RAM (bytes)	262,144	46,816 (17.9%)	47,096 (18%)
Flash (bytes)	983,040	370,912 (37.7%)	175,544 (17.9%)
Latency (ms)	/	322	47

**Table 8:** Overview of related work and this study.

Study	ME ± Stdev	Population and Methods
Hannink et al. [9] model A	−0.34 ± 8.10 cm	geriatric inpatients, walking overground, HS-HS, 10-fold cross-validation, stride length ground truth from the GAITRite.
Hannink et al. [9] model B	−0.15 ± 6.09 cm	geriatric inpatients, walking overground, HS-HS, 10-fold cross-validation, stride length ground truth from the GAITRite.
Hannink et al. [10]	0.27 ± 5.43 cm	geriatric patients walking overground, HS-HS, 10-fold cross-validation, stride length ground truth from the GAITRite.
Zrenner et al. [11]	1.3 ± 19.4 cm	amateur runners, running overground, HS-HS, leave-one-out cross-validation, stride length ground truth motion capture system
This study	1.07 ± 8.35 cm	healthy subjects, treadmill walking, HS-HS, leave-one-out cross-validation, stride length ground truth from the Optogait

## Data Availability

Not applicable.
